# Self-Administered Auricular Acupressure Integrated With a Smartphone App for Weight Reduction: Randomized Feasibility Trial

**DOI:** 10.2196/14386

**Published:** 2019-05-29

**Authors:** Lorna Suen, Wenru Wang, Kenneth King Yip Cheng, Matthew Chin Heng Chua, Jerry Wing Fai Yeung, Wai Kin Koh, Simon Kai Wang Yeung, Janice Yuen Shan Ho

**Affiliations:** 1 School of Nursing The Hong Kong Polytechnic University Hong Kong China (Hong Kong); 2 Alice Lee Centre for Nursing Studies, Yoog Loo Lin School of Medicine National University of Singapore Singapore; 3 Department of Health Technology and Informatics The Hong Kong Polytechnic University Hong Kong China (Hong Kong); 4 Smart Health Leadership Centre, Institute of Systems Science National University of Singapore Singapore

**Keywords:** acupressure, auriculotherapy, overweight, obesity, smartphone, leptin, adiponectin, randomized controlled trial

## Abstract

**Background:**

Obesity is a common global health problem and increases the risk of many chronic illnesses. Given the adverse effects of antiobesity agents and bariatric surgeries, the exploration of noninvasive and nonpharmacological complementary methods for weight reduction is warranted.

**Objective:**

The study aimed to determine whether self-administered auricular acupressure (AA) integrated with a smartphone app was more effective than using AA alone or the controls for weight reduction.

**Methods:**

This study is a 3-arm randomized waitlist-controlled feasibility trial. A total of 59 eligible participants were randomly divided into either group 1 (AA group, n=19), group 2 (AA plus smartphone app, n=19), or group 3 (waitlist control, n=21). A total of 6 reflective zones or acupoints for weight reduction were chosen. The smartphone app could send out daily messages to the subjects to remind them to perform self-pressing on the 6 ear acupoints. A “date picker” of the 8-week treatment course was used to enable the users to input the compliance of pressing and the number of bowel movement daily instead of using the booklet for recordings. The app also served as a reminder for the subjects regarding the dates for returning to the center for acupoint changing and assessments. Treatment was delivered 2 times a week, for 8 weeks. Generalized estimating equations were used to examine the interactions among the groups before and after intervention.

**Results:**

Subjects in group 2 expressed that the smartphone app was useful (7.41 out of 10). The most popular features were the daily reminders for performing self-pressing (88%), the ear diagram indicating the locations and functions of the 6 ear points (71%), and ear pressing method demonstrated in the video scripts (47%). Nearly 90% of the participants completed the 8-week intervention, with a high satisfaction toward the overall arrangement (8.37 out of 10). The subjects in group 1 and 2 achieved better therapeutic effects in terms of body weight, body mass index (BMI), waist circumference, and hip circumference and perceived more fullness before meals than the waitlist controls. Although no significant differences in the pairwise comparisons between the 2 groups were detected (*P*>.05), the decrease in body weight, BMI, body fat, visceral fat rating and leptin level, and increase in adiponectin level were notable in group 2 before and after the intervention.

**Conclusions:**

The high compliance rate and high satisfaction toward the trial arrangement indicate that AA can be used to achieve weight reduction and applied in future large-scale studies. AA integrated with the smartphone app has a more notable effect than using AA alone for weight reduction. Larger sample size should be considered in future trials to determine the causal relationship between treatment and effect.

**Trial Registration:**

ClinicalTrials.gov NCT03442712; https://clinicaltrials.gov/ct2/show/NCT03442712 (Archived by WebCite at http://www.webcitation.org/78L2tO8Ql)

## Introduction

### Background

Obesity is a common global health problem caused by different factors such as endocrine disorder, metabolic syndrome, improper diet, drugs, or heredity [1,2]. It increases the risk of many chronic illnesses, including but not limited to hypertension, type 2 diabetes mellitus, cardiovascular diseases, musculoskeletal disorders, sleep apnea, and certain types of cancer [2-5].

Conventional approaches to alleviate obesity include medications [4], exercise [6], dietary control [7], behavior modification therapy [8,9], or bariatric surgeries [10]. However, the safety of antiobesity agents is a concern because they may induce depressed mood disorders, anxiety, or even increased risk of suicide during treatment [4]. Gastric bypass surgery and other bariatric surgeries also pose potential risks such as excessive bleeding, bowel obstruction, dumping syndrome, hernias, or stomach perforation [11].

Auriculotherapy or auricular treatment involves stimulating points on the ear with sterile acupuncture needles or acupressure with magnetized pellets or seeds [12]. Auriculotherapy offers a more effective and economical option to treat obesity than conventional approaches [13]. Auricular acupuncture has been frequently used to treat obesity in many countries [14-17]. However, the use of needles for auricular acupuncture may not be acceptable to people with needle phobia and could pose a risk of blood-borne transmission through needle prick injuries. Other methods of auricular acupoint stimulation include the application of electroacupuncture [18,19] or auricular acupressure (AA) using magnetic pellets or seeds [17-20]. Some researchers [21,22] attempted to adopt a combined approach by integrating auriculotherapy with diet restriction. Although auriculotherapy was found to be effective for weight reduction and management of dyslipidemia, the effect solely attributed to auriculotherapy could not be determined.

AA is a safe, noninvasive, inexpensive, and easily self-administered approach that causes few adverse effects [13,15,17]. A number of studies have attempted to examine the efficacy of using different materials for AA in weight reduction. The authors [13,15] concluded that using *semen vaccariae* is more effective than magnetic pellets for lowering body weight. During AA treatment, self-administered pressing on the seeds by the patients is necessary to achieve adequate acupoint stimulation. However, previous therapists may have difficulties in monitoring the compliance of subjects to perform seed pressing [17,21,23,24] that might affect the intervention dosages. Some controversial findings have indicated that AA does not significantly change the anthropometric parameters between experimental and control groups [25] probably because of a low compliance rate of subjects in conducting seed pressing, which the researchers fail to monitor.

Smartphones are currently the most popular communication tools, with approximately 70% of the global population using them. Accelerometer-based tracking devices and smartphone apps have been increasingly used to promote health because of their potential to influence self-regulation of a person’s behavior [26-28].

Given that weight reduction increases the serum adiponectin concentration, resulting in a decrease in leptin levels [29], outcome measures should not only include anthropometric indices but also hormonal changes (leptin concentration and adiponectin level) associated with weight reduction. Determining the changes in plasma leptin and adiponectin levels could facilitate our understanding of the association of these biomarkers and the underlying mechanisms of the treatment protocol on weight reduction. A smartphone app (namely ‘Auricular Acupressure for Weight Reduction, V1’) was developed in this trial to monitor and enhance subjects’ compliance on performing pressings to the acupoints.

### Objectives

Our work aims to determine whether self-administered AA integrated with smartphone app was more effective than using AA alone or the controls for weight reduction. Subjects’ satisfaction level toward the treatment protocol and the smartphone app, the recruitment and attrition rate of the subjects were evaluated. The preliminary effects of the treatment protocols, the hormonal changes associated with weight reduction, and the effect sizes of the treatment protocols were also determined. The findings of this feasibility study could provide valuable information for future large-scale studies.

## Methods

### Settings and Participants

This study is a 3-arm randomized waitlist-controlled feasibility trial. On the basis of the previous studies [20,30] of the effectiveness of AA on weight reduction, a medium effect size is considered for sample size calculation. According to Whitehead et al [31], a sample size of 15 per treatment arm is adequate. Considering an attrition rate of 20%, 19 to 20 subjects per arm were recruited for this feasibility study.

Subjects who met the following inclusion criteria were recruited from the community through snowball sampling via social network platforms (WhatsApp and Facebook): (1) age 18 years or older; (2) overweightness, with body mass index (BMI) ≥25.0 kg/m^2^ in accordance with the BMI classification of the World Health Organization (WHO) [2]; (3) neither received other weight control measures nor experienced medical or drug history within the last 3 months; (4) no ear injuries, such as inflammation or lesions, and no medical history of ear surgery within the last 6 months; and (5) smartphone user. Exclusion criteria were (1) diabetes, severe hypertension, heart disease or endocrine abnormalities; (2) pregnancy; (3) SCOFF (a questionnaire utilizing an acronym in 5 simple questions on “Sick, Control, One stone (6.5 kg), Fat, and Food”) score ≥2 out of 5 items, which indicates eating disorders [14]; and/or (4) psychiatric and mental disorders.

### Groupings and Procedure

**Figure 1:**
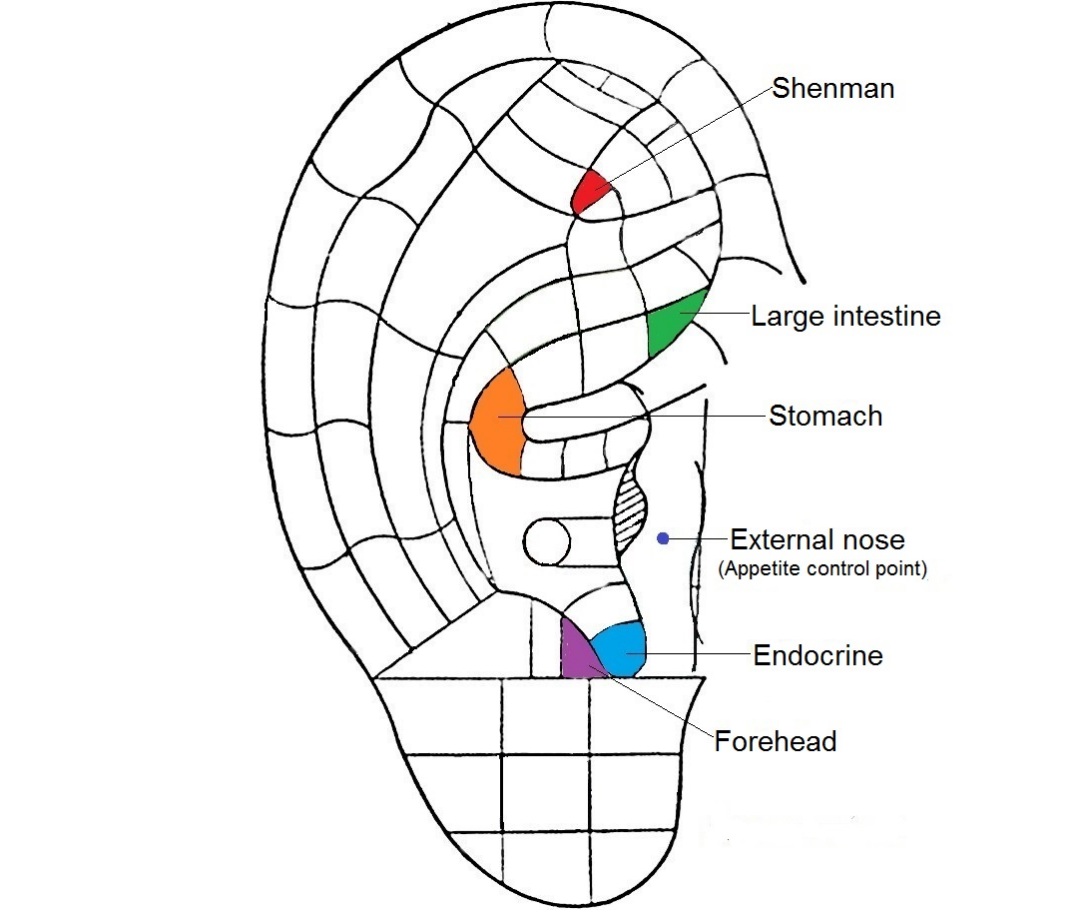
Ear acupoints for weight reduction.

Eligible subjects were randomly allocated to 1 of the 3 groups by using a computer-generated randomized table. The random allocation sequence was placed in an opaque, sequentially numbered, sealed envelope to guarantee adequate allocation concealment. The therapy was administered by a researcher (SY) who had received intensive coaching by the research team (LS, JY), and reliability on the accuracy of ear point identification was established. A total of 6 reflective zones or acupoints for weight reduction were chosen. These acupoints included “shenmen” (TF_4_), “stomach” (CO_4_), “endocrine” (CO_18_), “external nose” (TG_1, 2i_), “large intestine” (CO_7_), and “forehead” (AT_1_). The Chinese Standard Ear Acupoints system [32,33] and the nomenclature of the Nogier auricular acupoints (European system) [34,35] were taken as reference for acupoint selection and location identification (Figure 1). The principles of acupoint selection are to reduce excessive calorie intake and promote waste excretion to achieve weight reduction. The research team, which included an academic with over 20 years of research experience on auriculotherapy (LS) and 2 team members registered as traditional Chinese medicine practitioners of Hong Kong (JY and JH), selected the acupoints to be used.

#### Group 1: Auricular Acupressure Only

**Figure 2:**
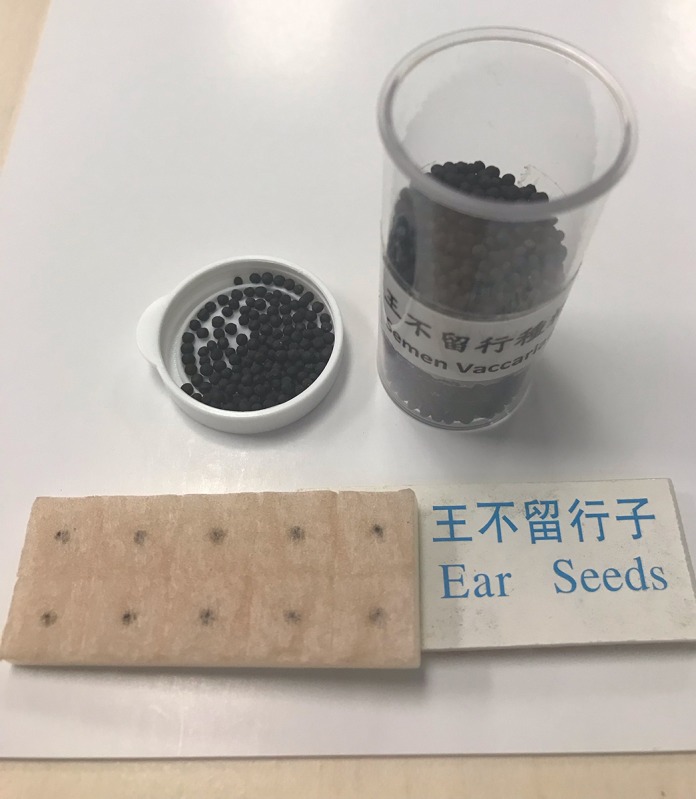
Semen vaccariae for auricular acupressure.

AA treatment using *semen vaccariae* seeds [36] was performed by the researcher. The seeds were kept in place by a piece of adhesive tape (Figure 2) and were applied on 1 ear only. The researcher met with the subjects twice weekly to change the tapes every 3 to 4 days to the opposite ear to prevent skin irritation. Subjects were requested to apply pressure 20 times using a constant rhythm to each point thrice per day, preferably within 30 min before meals. Coaching on how to self-administer AA on the acupoints was given to the subjects, and a return demonstration was required to ensure that the treatment was performed properly. An information booklet containing acupoint location and functions and the self-pressing methods was given to the subjects. The subjects were requested to record the frequency of daily pressing and bowel movement per day and show their records to the researcher in every visit for checking purposes.

#### Group 2: Auricular Acupressure Plus Smartphone App

**Figure 3:**
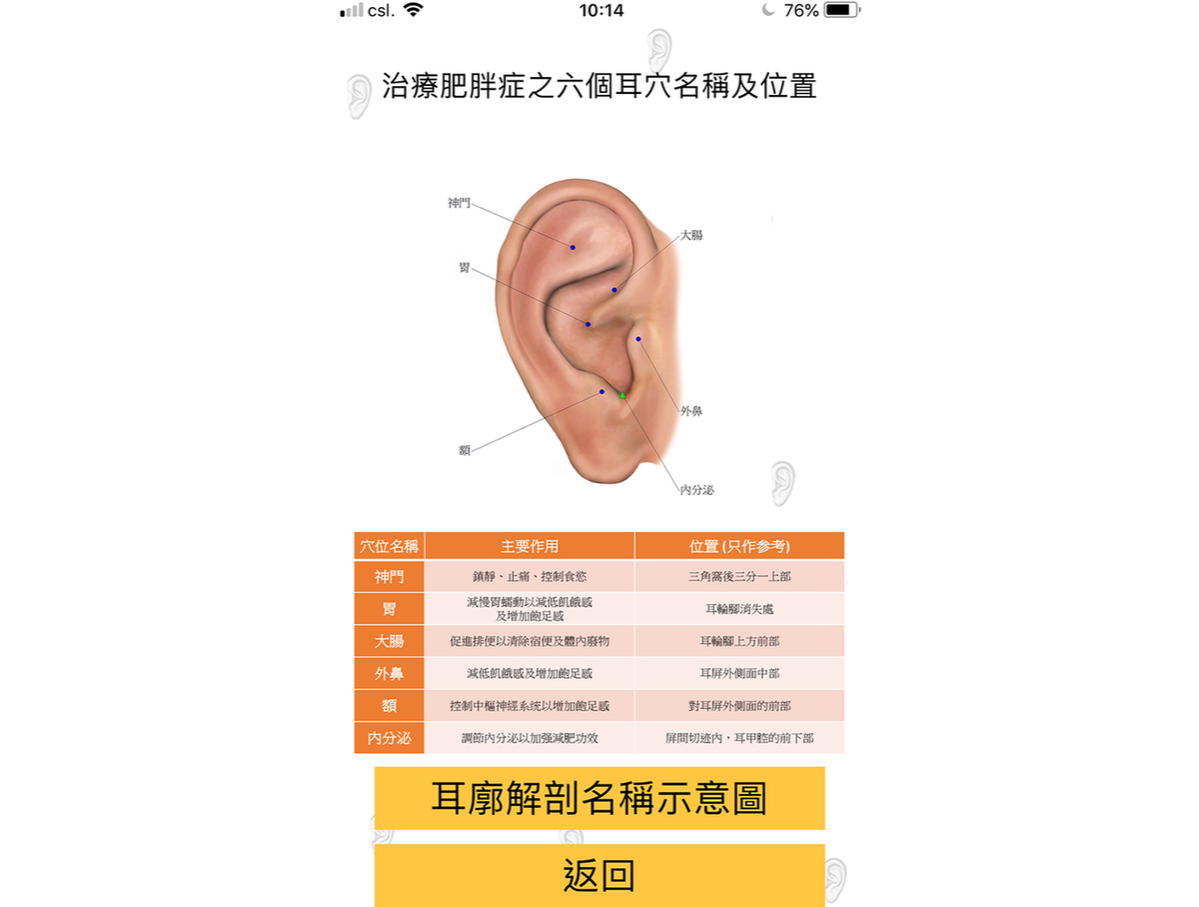
An ear diagram indicating the locations and functions of the 6 ear points in the app.

**Figure 4:**
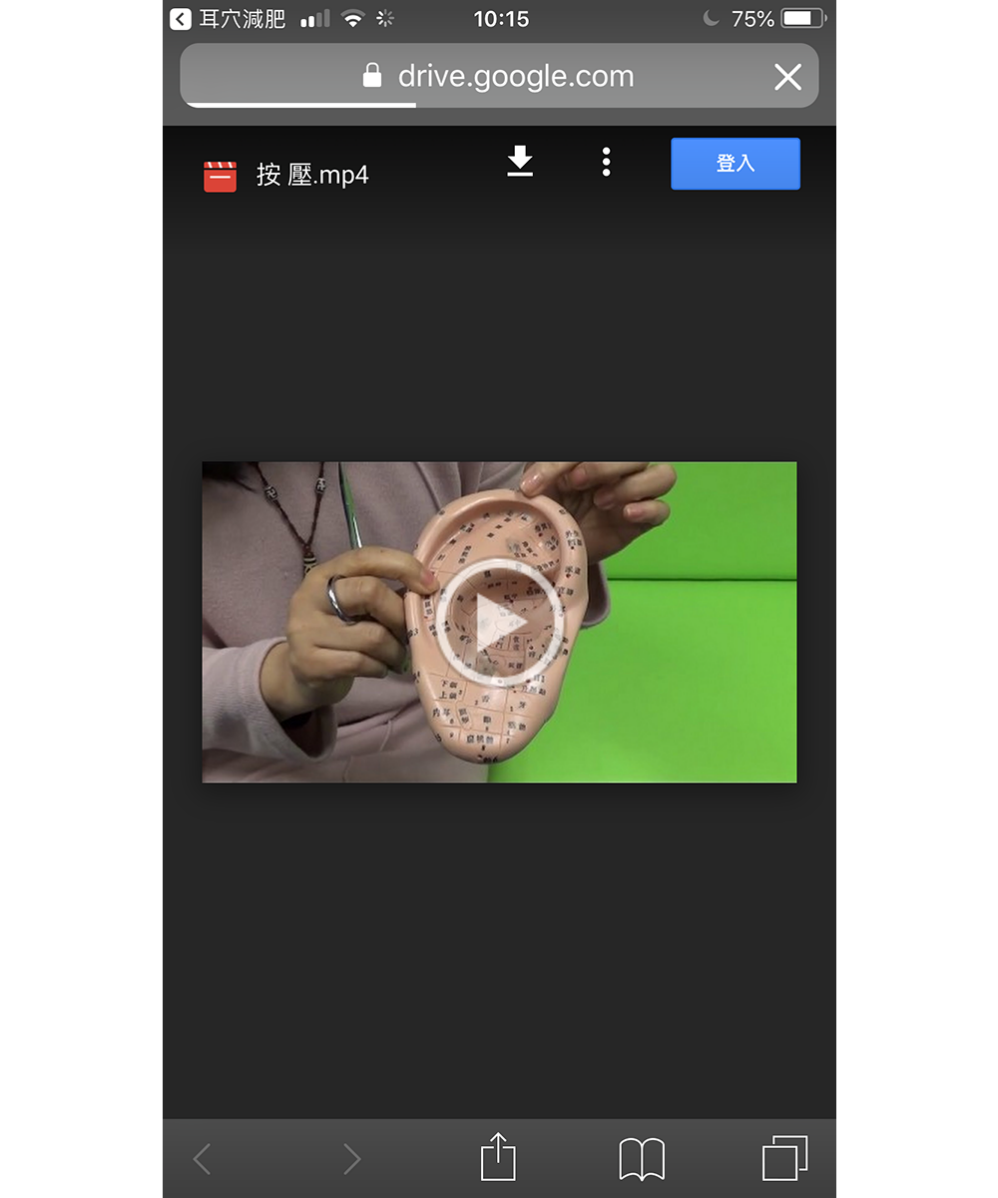
Video script that demonstrated proper ear pressing method in the app.

**Figure 5:**
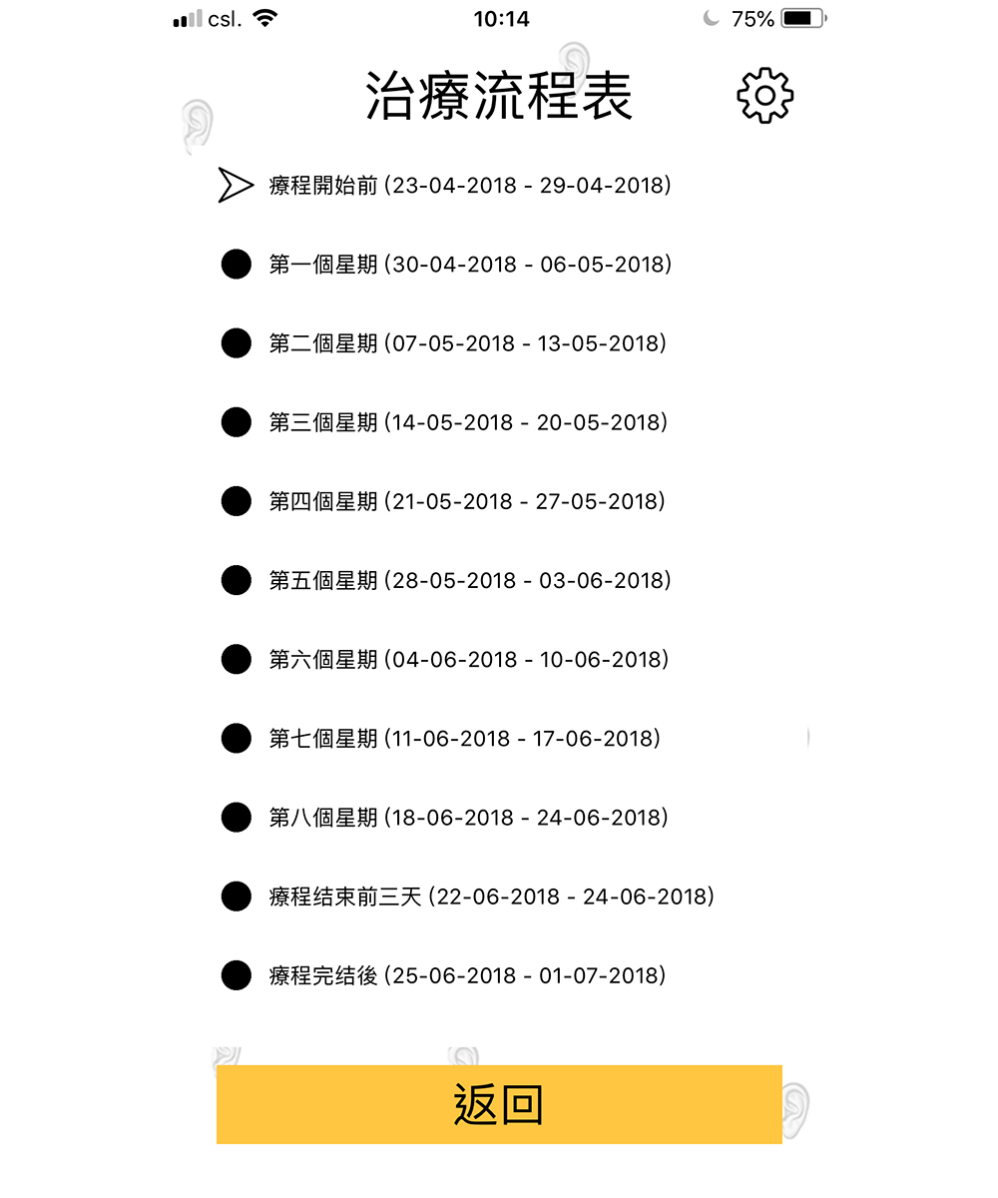
The eight-week treatment schedule was populated automatically once the user selected the starting date in the app.

**Figure 6:**
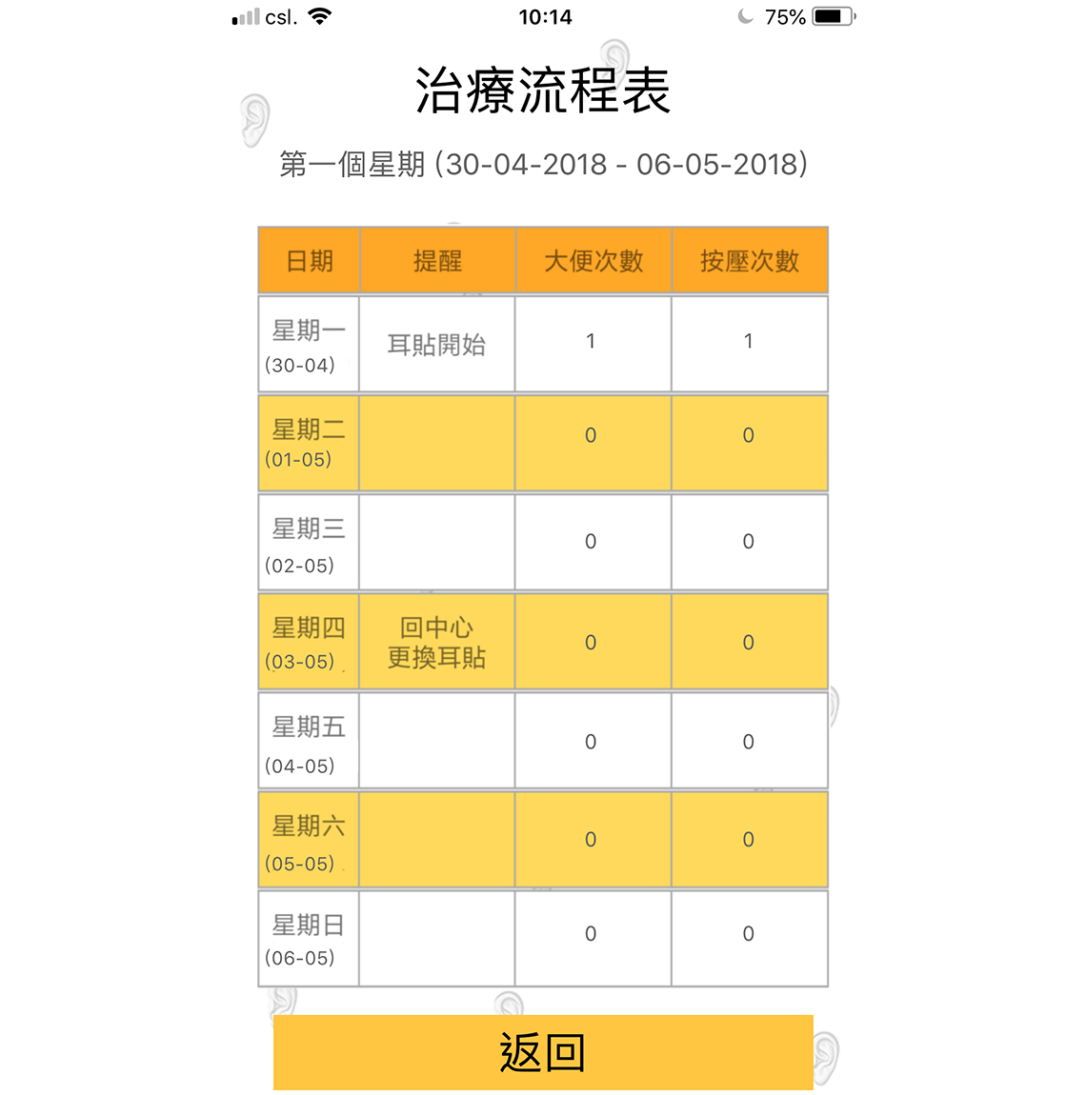
The weekly schedule list which allows the user to input the compliance of pressing and the number of bowel movement daily in the app.

A smartphone app, namely “Auricular Acupressure for Weight Reduction, V1,” which is applicable for iPhone operating system (iOS) and android users and was written in Chinese, was developed (Figures 3-6). To obtain better performance and improve user experience, the smartphone app was developed natively by using Java (Version 8.0, Oracle Corporation) for Android phone user, and Swift (Version 4.0, Apple Inc) for iPhone user. Apart from providing the AA treatment and information booklet for the subjects, the developed app was installed in the smartphone of the subjects. The app contains an ear diagram indicating the locations and functions of the 6 ear points, video scripts that demonstrated proper ear pressing method, and precautions for performing AA. To minimize the size of the app, the video scripts were stored in the cloud, and the video was streamed on the fly. During the first launch of the app, a “date picker” prompted the user to choose the starting date of the treatment. Once the user selected the starting date, it was verified in the background, and the 8-week treatment schedule was populated automatically as shown in Figure 5. Once user clicked on the schedule, it directed the user to the weekly schedule list where the user would be able to input the compliance of pressing and the number of bowel movement daily (Figure 6) instead of using the booklet for recordings. The data were then cached in the phone. The app sent out daily notifications to the subjects to remind them to perform self-pressing on the 6 ear acupoints. An algorithm based on the decision tree method was embedded in the app to determine the number of reminders and content of the notification. The frequency of sending reminders to the subjects by the app was accorded with the subjects’ compliance of self-pressing. Normally, only 1 reminder/day was sent to the subject if they indicated good compliance (ie, 2-3 pressings/day), and an additional reminder would be sent to them the next day if the compliance is poor (ie, 0-1 time/day). In response to subjects’ compliance on acupoint pressing, some messages such as “Excellent job” for those with good compliance and “You are almost there, keep going” for those with poor compliance would “pop up” upon data entry as positive reinforcement. The app also served as a reminder for the subjects regarding the dates for returning to the center for acupoint changing and assessments. In case the subjects encountered any difficulties during the process, they could send out a message to the researcher for receiving timely advice via the app. More details about the screenshots (in Chinese) of the “Auricular Acupressure for Weight Reduction, V1” app are shown in Multimedia Appendix 1.

#### Group C: Waitlist Control Group

The subjects in the waitlist control group were told to maintain their usual dietary and exercising patterns during the waiting period. They were also required to receive the assessments similar to subjects in the other 2 groups. AA treatment plus smartphone app was given to these subjects after the 8-week intervention of the 2 experimental groups.

The following procedures were standardized for the subjects in groups 1 and 2. The auricle of the subject was cleaned using 75% isopropyl alcohol before therapy administration. Only 1 ear received treatment at a time. The experimental objects (seeds) were applied to the reactive region of these acupoints indicated by an acupoint finder (Pointer Plus) [37]. Treatment was firstly applied to the right ear during the first visit, followed by the left ear during the second visit, and so on. The experimental objects were replaced twice weekly to avoid local irritation of the auricular points under treatment. The total treatment period lasted for 8 weeks.

Ethical approval was obtained from the Human Research Ethics Review Committee of the Hong Kong Polytechnic University. The study was conducted in accordance with the Declaration of Helsinki. Participation in the study was voluntary. Written informed consent was obtained from each subject upon explanation of the risks and benefits of their participation. Given the multiple visits to the centers for receiving the protocol, a travel subsidy in the form of supermarket coupons (approximately US $25) was given to each subject upon completion of the study.

### Outcome Measures

Another assistant who was unaware of the type of treatment modality received by the subjects evaluated the effect of treatment to achieve evaluator blinding. All outcome measurements were conducted at baseline and post intervention at 8 weeks. The BMI (kg/m^2^) was taken as the primary outcome of the study. Secondary outcomes included other anthropometric indices such as body weight (kg), body fat (%), body water (%), and visceral fat rating, which were determined by a body composition analyzer (Model: Tanita BC-545N) [38]. Waist (cm) and hip circumferences (cm) were taken twice using standard method [39] to ensure accuracy.

Laboratory tests for leptin and adiponectin testing were conducted via standardized methods. Leptin concentration was measured according to the manufacturer’s instructions using a commercial sandwich ELISA kit [40] comprising ready-to-use components, which were either concentrated or lyophilized. The dilution factor was considered during the calculation of leptin concentrations. The limit of detection of the leptin assay was 0.2 ng/mL. The research team would repeat the tests if results exceeded a leptin concentration of 50 ng/mL for diluted samples. Precision intra-assay (within-run), coefficient of variability (CV)=5.9%, and inter-assay (run-to-run) CV=5.6% were reported to express the precision or repeatability of the immunoassay test result [5]. Adiponectin level was measured with a commercially available sandwich ELISA kit [41]. The assay has a sensitivity of 1.5 ng/mL^−1^. The serum concentrations of leptin and adiponectin were calculated based on standard curves plotted according to the manufacturer’s instructions [29].

Subjects were asked to rate the fullness level before lunch and dinner using a visual analog scale of 0 to 10 (adapted from Rock et al) [42] for 3 consecutive days at baseline and post intervention. The question was “How full do you feel?,” with anchor values ranging from 0 (“Not at all full”) to 10 (“Totally full”). Before the therapy, the subjects were asked regarding their confidence and perceived usefulness of the treatment to manage their overweight problem. Upon completion of the 8-week protocol, the satisfaction of the subjects toward the therapy, the information booklet, and the smartphone app (if applicable) was also evaluated using a 10-point scale, with higher scores indicating greater satisfaction.

### Data Analyses

Descriptive statistics for sociodemographic characteristics of the subjects were presented. The estimated mean and SE of the outcome variables before and after intervention were computed. The association between categorical variables was examined using χ^2^ test or Fisher exact test. The Mann–Whitney *U* test or Kruskal–Wallis test was used for detecting group differences where appropriate.

Primary analysis was conducted using the generalized estimating equations with an auto-regression correlation structure to examine the interactions among the groups before and after intervention on the primary and secondary outcome variables, including anthropometric indices, perceived hunger level before meals, leptin concentration (ng/mL), and adiponectin level (ng/mL). Missing data were addressed using the GEE model and assumed to be missed at random [43]. Apart from conducting analyses on all participants, we repeated the main analysis of the completers who had finished the treatment protocol for sensitivity analysis. Correlation analyses among the anthropometric indices and the biomarkers were conducted. The reported adverse effects, expectation, and satisfaction toward the therapy were evaluated. We used SPSS version 25.0 (IBM Corporation) for all statistical analyses. All statistical tests were 2-sided, with the significance level set to 0.05.

## Results

The data were collected from April to November 2018. The recruitment rate was fairly high (72%). Within 2 weeks of promotion via the social platforms (WhatsApp and Facebook), 82 enquiries regarding the project were received. A total of 59 eligible participants were randomly divided into 3 groups (group 1=19, group 2=19, and group 3=21). Participants who were excluded were mainly because of having metabolic syndrome with uncontrolled diabetic or hypertensive conditions. After 8 weeks of intervention, the attrition rate was only 10% (n=6). The flow diagram of the participants of this trial is illustrated in Multimedia Appendix 2. The trial is reported in accordance with Consolidated Standards of Reporting Trials of Electronic and Mobile HEalth Applications and onLine TeleHealth in Multimedia Appendix 3.

### Subject Characteristics

The recruited subjects had an average age of 49.15 years (SD 10.54), with a mean BMI of 30.35 (SD 4.53 kg/m^2^). The waist–hip ratio was 0.95 (SD 0.63) and 0.90 (SD 0.05) for males and females, respectively. Males only accounted for 15% (n=9) of the subjects. The groups were essentially comparable and well balanced in age, gender distribution, BMI, education level, marital status, comorbid illnesses, exercising level, daily fluid intake, smoking status, and alcohol consumption. Majority of the participants had eating-out habits at least once per day (83%), perceived themselves as a gluttonous person (48%), ever attempted other means of weight reduction (58%), and worried about their overweight problem. Women significantly felt unhappier regarding their overweight problem than men (*P*<.05). None of the participants had eating disorders as verified by the SCOFF questionnaire [14]. Among the subjects, there were more android users (37/59, 63%) than iOS users (22/59, 37%; Table 1).

**Table 1:** Sociodemographic and baseline characteristics of the subjects (N=59).

Characteristics		All	Group 1: AA^a^ (n=19)	Group 2: AA + app (n=19)	Group 3: Waitlist control (n=21)	*P* value
Age (years), mean (SD)		49.15 (10.54)	47.58 (11.59)	49.21 (9.70)	50.52 (10.58)	.87^b^
**Gender, n (%)**						
	Male	9 (15)	4 (21)	3 (16)	2 (10)	.60^c^
	Female	50 (85)	15 (79)	16 (84)	19 (90)	—^d^
**Education level, n (%)**						
	Primary or below	1 (2)	0 (0)	1 (5)	0 (0)	.36^c^
	Secondary	32 (54)	13 (68)	8 (42)	11 (52)	—
	Tertiary or above	26 (44)	6 (32)	10 (53)	10 (48)	—
**Marital status, n (%)**						
	Single, divorced/widowed	19 (32)	10 (53)	5 (26)	4 (19)	.08^c^
	Married	40 (68)	9 (47)	14 (74)	17 (81)	—
Body mass index (kg/m^2^), mean (SD)		30.35 (4.53)	30.32 (4.65)	30.65 (5.41)	30.11 (3.70)	.97^b^
**Waist-hip ratio, mean (SD)**						
	Male	0.95 (0.63)	0.97 (0.59)	0.91 (0.08)	0.97 (0.06)	.04^b^
	Female	0.90 (0.05)	0.91 (0.05)	0.89 (0.05)	0.89 (0.05)	.25^b^
**Comorbid illness, n (%)**						
	No	37 (63)	10 (53)	12 (63)	15 (71)	.48^e^
	Yes	22 (37)	9 (47)	7 (37)	6 (29)	—
**Regular drugs taken, n (%)**						
	No	39 (66)	11 (58)	13 (68)	15 (71)	.70^e^
	Yes	20 (34)	8 (42)	6 (32)	6 (29)	—
**Regular exercise, n (%)**						
	No	30 (51)	10 (53)	8 (42)	12 (57)	.72^e^
	Yes	29 (49)	9 (47)	11 (58)	9 (43)	—
**Smoking habit, n (%)**						
	Never	56 (95)	16 (84)	19 (100)	21 (100)	.06^c^
	Ex-smoker	2 (3)	2 (11)	0 (0)	0 (0)	—
	Current smoker	1 (2)	1 (5)	0 (0)	0 (0)	—
**Drinker, n (%)**						
	Never	21 (36)	7 (37)	7 (37)	7 (33)	>.99^e^
	Social drinker	38 (64)	12 (63)	12 (63)	14 (67)	—
Cups of fluid intake/day, mean (SD)		6.84 (2.04)	6.63 (2.41)	6.84 (1.73)	7.05 (2.02)	.82^b^
**Number of times/day eat-out, n (%)**						
	0	10 (18)	3 (16)	4 (21)	3 (16)	.42^c^
	1-2	37 (64)	13 (68)	13 (68)	11 (58)	—
	3-4	10 (18)	3 (16)	2 (11)	5 (26)	—
**Claim to be a gluttonous person, n (%)**						
	Sometimes	31 (53)	14 (74)	7 (37)	10 (48)	.05^e^
	Always	28 (47)	5 (26)	12 (63)	11 (52)	—
**Bowel habit, n (%)**						
	Regular bowel open	43 (73)	12 (63)	13 (68)	18 (86)	.36^c^
	Occasion constipation	11 (19)	4 (21)	4 (21)	3 (14)	—
	Frequent constipation	5 (8)	3 (16)	2 (11)	0 (0)	—
**Ever try other means for weight reduction, n (%)**						
	No	25 (42)	7 (37)	8 (42)	10 (48)	.76^e^
	Yes	34 (58)	12 (63)	11 (58)	11 (52)	—
**Worry about overweight, n (%)**						
	Always	17 (29)	6 (32)	7 (37)	4 (19)	.23^c^
	Sometimes	34 (58)	8 (42)	11 (58)	15 (71)	—
	Never	8 (13)	5 (26)	1 (5)	2 (10)	—
**Unhappy owing to overweight, n (%)**						
	Always	10 (17)	5 (26)	4 (21)	1 (5)	.29^c^
	Sometimes	33 (56)	8 (42)	10 (53)	15 (71)	—
	Never	16 (27)	6 (32)	5 (26)	5 (24)	—
**Phone model, n (%)**						
	Android	37 (63)	9 (47)	13 (68)	15 (71)	.29^e^
	iPhone operating system	22 (37)	10 (53)	6 (32)	6 (29)	—
Sick, Control, One stone (6.5 kg), Fat, and Food score, mean (SD)		0.32 (0.80)	0.47 (1.17)	0.37 (0.68)	0.14 (0.36)	.52^b^

^a^AA: auricular acupressure.

^b^Kruskal-Wallis test.

^c^Fisher exact test.

^d^Not applicable.

^e^Chi-square test.

### Compliance, Expectation, and Satisfaction Toward Treatment

The compliance to the intervention protocol was high, with an average of 90% (n=53) having completed the 8-week intervention. Even though over 76% of the subjects did not experience receiving complementary and alternative treatment, they generally exhibited a positive attitude toward AA (6.53 out of 10) before the trial. After the intervention, their satisfaction with the overall arrangement was high (8.37 out of 10). The compliance for performing self-pressing was satisfactory, with 79% of subjects in groups 1 and 2 performing self-pressing 3 times or more before meals throughout the protocol. No significant difference in the pressing compliance and frequency of bowel movement between groups was noted.

Subjects in group 2 expressed that the smartphone app was useful (7.41 out of 10). The most popular features were the daily reminders for performing self-pressing (88%), the ear diagram indicating the locations and functions of the 6 ear points (71%), and ear pressing method demonstrated in the video scripts (47%). Majority of the subjects in groups 1 and 2 (n=34, 97%) indicated that they would consider recommending this therapy to others. No specific adverse effects arising from the therapy were observed, except 2 participants (5%) who reported having mild skin irritation on the ears because of the adhesive tapes for holding the experimental objects in place. The most tender acupoint felt by the subjects was “stomach” (74%), followed by “forehead” (34%), “endocrine” (32%), “external nose” (18%), “shenmen” (16%), and “large intestine” (16%). Only 2 male subjects (6%) felt embarrassed because of the adhesive tapes put on the auricles (Table 2).

**Table 2:** Reported adverse effects, expectations, and satisfaction toward the therapy (N=59).

Variables		All	Group 1: AA^a^ (n=19)	Group 2: AA + app (n=19)	Group 3: Waitlist control (n=21)	*P* value
**Have you used complementary therapies in the past?^b^**						
	No	45 (76%)	16	14	15	.65^c^
	Yes	14 (24%)	3	5	6	—^d^
How much confidence do you have in complementary therapies in general (0-10)^b^		6.05 (1.91)	5.84 (1.89)	6.05 (2.17)	6.24 (1.73)	.93^e^
Perceived usefulness of the treatment being received(0-10)^b^		6.53 (1.52)	6.16 (1.54)	6.68 (1.34)	6.71 (1.68)	.37^e^
**What will you expect about the overweight problem after 8 weeks (post intervention)?^b^**						
	Totally resolve	0	0	0	0	.28^c^
	Improve greatly	9	3	2	4	—
	Moderate improvement	22	10	6	6	—
	Little improvement	20	3	10	7	—
	Same as before	8	3	1	4	—
Ear itchiness^f^		2 (5%)	2	0	—	—
**Tenderness on acupoints^f^**						
	Shenmen	6 (16%)	2	4	—	—
	Stomach	28 (74%)	14	14	—	—
	External nose	7 (18%)	4	3	—	—
	Forehead	13 (34%)	7	6	—	—
	Large intestine	6 (16%)	5	1	—	—
	Endocrine	12 (32%)	7	5	—	—
**Change in satiety level^f^**						
	Decrease appetite	23 (62%)	12	10	—	.20^c^
	No change	12 (32%)	5	7	—	—
	Increase appetite	2 (5%)	1	0	—	—
**Compliance on performing pressing on acupoints^f^**						
	<3 times/day	7	4	3	—	>.99^c^
	3 times/day	21	11	10	—	—
	>3 times/day	9	4	5	—	—
Satisfaction toward the overall arrangement (0- 10)^f^		8.37 (1.68)	8.61 (1.85)	8.12 (1.50)	—	.18^e^
Satisfaction toward the treatment effect (0-10)^f^		5.63 (2.46)	5.89 (2.25)	5.35 (2.71)	—	.65^e^
Usefulness of the information booklet^f^, mean (SD)		6.03 (2.53)	7.00 (2.30)	5.00 (2.40)	—	.02^e^
Usefulness of the mobile app^f^, mean (SD)		—	—	7.41 (2.53)	—	—
**Usefulness of the features in the app^f^**						
	Reminder	—	—	15 (88%)	—	—
	Video script	—	—	6 (36%)	—	—
	Contact the researcher	—	—	4 (24%)	—	—
	Ear diagram	—	—	12 (71%)	—	—
	Ear pressing method demonstration	—	—	8 (47%)	—	—
**Will recommend this therapy to others^f^**						>.99^e^
	Definitely will	10 (29%)	5	5	—	—
	Maybe	24 (69%)	12	12	—	—
	No	1 (3%)	1	0	—	—
**Embarrass owing to ear plaster^f^**						
	No	33	18	15	—	.23^e^
	Yes	2	0	2	—	—

^a^AA: auricular acupressure.

^b^Evaluated before the intervention.

^c^Fisher exact test.

^d^Not applicable.

^e^Mann-Whitney *U* test or Kruskal-Wallis test as appropriate.

^f^Evaluated after the intervention has been completed.

### Treatment Effect

In general, the subjects in groups 1 and 2 achieved better therapeutic effects in terms of body weight (kg), BMI (kg/m^2^), waist circumference (cm), and hip circumference (cm), and perceived more fullness before meals than the waitlist controls. Although no significant differences in the pairwise comparisons between the 2 groups were detected, the decrease in body weight, BMI, body fat, visceral fat rating and leptin level, and increase in adiponectin level were notable in group 2 before and after the intervention (Table 3). When the effect size was estimated using the primary outcome (BMI), a medium effect size (*d*=0.4928) was determined in group 1 (AA), whereas a large effect size (*d*=0.7798) was detected in group 2 (AA plus app), taking the waitlist control group as reference. Correlation analyses indicate that significant correlations were present among the anthropometric indices and the leptin concentration (ng/mL) after the intervention (Multimedia Appendix 4). A completer’s analysis showed consistent findings on the outcome variables of the trial.

**Table 3:** Outcome variables across the 3 groups before and after intervention.

Measures		Grouping						Pairwise comparisons between groupsBeta (95% CI)		
		Group 1: AA^a^ (n=19)		Group 2: AA + app (n=19)		Group 3: Waitlist control (n=21)		Group 1 versus group 2	Group 1 versus group 3	Group 2 versus group 3			
		Estimated mean (SE)	Mean difference (*P* value)	Estimated mean (SE)	Mean difference (*P* value)	Estimated mean (SE)	Mean difference (*P* value)			
**Body weight (kg)**										
	Baseline	78.87 (2.83)	1.33 (0.005)	78.00 (3.15)	1.56 (0.000)	75.68 (2.18)	0.23 (0.515)	–0.23 (–1.38 to 0.92)	1.10 –0.07 to 2.26)	1.33 (–0.36 to 2.29)
	Postintervention	77.54 (2.63)	—^b^	76.44 (3.09)	—	75.44 (2.12)	—	—	—	—
**Body mass index (kg/m^2^** **)**										
	Baseline	30.32 (1.04)	0.50 (0.013)	30.65 (1.21)	0.60 (0.000)	30.11 (0.79)	0.12 (0.435)	–0.10 (–0.56 to 0.36)	0.38 (–0.12 to 0.87)	0.48 (0.09 to 0.87)
	Postintervention	29.82 (0.98)	—	30.05 (2.21)	—	29.99 (0.75)	—	—	—	—
**Body fat (%)**										
	Baseline	39.58 (1.72)	0.17 (0.640)	40.73 (2.18)	0.39 (0.012)^c^	41.34 (1.44)	0.66 (0.274)	–0.23 (–1.00 to 0.54)	–0.49 (–1.88 to 0.89)	–0.27 (–1.49 to 0.96)
	Postintervention	39.42 (1.71)	—	40.33 (2.19)	—	40.68 (1.51)	—	—	—	—
**Body water (%)**										
	Baseline	45.21 (1.04)	–0.33 (0.190)	44.66 (1.33)	0.23 (0.144)	44.83 (0.89)	0.17 (0.641)	–0.56 (–1.15 to 0.02)	–0.51 (–1.38 to 0.37)	0.06 (–073 to 0.84)
	Postintervention	45.54 (1.06)	—	44.43 (1.31)	—	44.66 (0.84)	—	—	—	—
**Visceral fat rating**										
	Baseline	11.05 (0.85)	0.28 (0.021)	11.13 (0.64)	0.62 (0.066)	10.45 (0.57)	–0.28 (0.477)	–0.34 (–1.04 to 0.36)	0.56 (–0.25 to 1.36)	0.90 (–0.12 to 1.92)
	Postintervention	10.78 (0.80)	—	10.51 (0.61)	—	10.73 (0.62)	—	—	—	—
**Waist circumference (cm)**										
	Baseline	101.08 (2.61)	2.81 (0.001)	98.85 (2.55)	1.60 (0.006)	97.65 (1.54)	–0.76 (0.185)	1.21 (–0.79 to 3.21)	3.57 (1.59 to 5.55)	2.36 (0.75 to 3.96)
	Postintervention	98.27 (2.31)	—	97.25 (2.52)	—	98.41 (1.51)	—	—	—	—
**Hip circumference (cm)**										
	Baseline	109.66 (2.29)	1.53 (0.003)	110.74 (2.36)	1.41 (0.002)	109.07 (1.57)	–0.26 (0.612)	0.12 (–1.23 to 1.46)	1.78 (–2.55 to –0.51)	1.67 (0.35 to 2.99)
	Postintervention	108.14 (2.19)	—	109.33 (2.42)	—	109.32 (1.48)	—	—	—	—
**Waist-to-hip ratio**										
	Baseline	0.92 (0.13)	0.01 (0.084)	0.89 (0.01)	0.00 (0.694)	0.90 (0.01)	0.00 (0.259)	0.01 (–0.01 to 0.03)	0.02 (0.00 to 0.03)	0.01 (–0.01 to 0.02)
	Postintervention	0.91 (0.01)	—	0.89 (0.01)	—	0.90 (0.01)	—	—	—	—
**Leptin (ng/ml)**										
	Baseline	63.87 (9.97)	1.98 (0.850)	77.81 (14.23)	12.11 (0.243)	70.05 (9.50)	14.24 (0.071)	–10.13 (–38.98 to 18.71)	–12.27 (–37.93 to 13.39)	–2.14 (–27.67 to 23.40)
	Postintervention	61.90 (9.14)	—	65.70 (11.19)	—	55.80 (6.32)	—	—	—	—
**Adiponectin (ng/ml)**										
	Baseline	14896.48 (2190.72)	–370.60 (0.671)	16163.83 (2671.44)	–1468.54 (0.717)	14117.40 (2063.87)	–239.12 (0.876)	1097.94 (–7036.93 to 9232.82)	–131.47 (0.36 to 3321.20)	–1229.42 (–9730.36 to 7271.53)
	Postintervention	15267.08 (2309.31)	—	17632.37 (3384.36)	—	14356.52 (1846.75)	—	—	—	—
**Fullness (lunch)**										
	Baseline	4.54 (0.58)	–0.60 (0.333)	4.51 (0.47)	–1.06 (0.047)^c^	6.02 (0.50)	–0.28 (0.399)	0.82 (–1.15 to 2.07)	–0.32 (–1.70 to 1.06)	1.06 (0.01 to 2.12)^c^
	Postintervention	5.15 (0.44)	—	5.57 (0.43)	—	6.30 (0.53)	—	—	—	—
**Fullness (dinner)**										
	Baseline	4.60 (0.62)	–0.71 (0.321)	4.51 (0.51)	–1.40 (0.010)	6.76 (0.51)	0.26 (–0.328)	–0.69 (–1.06 to 2.45)	–0.97 (–2.45 to 0.52)	1.40 (033 to 2.47)
	Postintervention	5.30 (0.47)	—	5.91 (040)	—	6.50 (0,59)	—	—	—	—

^a^AA: auricular acupressure.

^b^Not applicable.

^c^Estimated mean and standard error (SE) from generalized estimating equations.

## Discussion

### Principal Findings

The findings of this 3-arm randomized waitlist-controlled study on 59 eligible participants demonstrates that the trial arrangement was feasible. The high compliance rate and high satisfaction toward the study indicate that AA can be used to achieve weight reduction and applied in future large-scale studies. Although no significant differences in the pairwise comparisons between AA integrated with the smartphone app (group 2) and AA only (group 1) were detected, the decrease in body weight, BMI, body fat, visceral fat rating, and leptin level and increase in adiponectin level were notable in group 2 before and after the intervention. The most popular features of the smartphone app were the daily reminders for performing self-pressing, the ear diagram indicating the locations and functions of the 6 ear points, and ear pressing method demonstrated in the video scripts. Majority of the subjects receiving AA treatment indicated that they would consider recommending this therapy to others. No specific adverse effects arising from the therapy were observed.

The high recruitment rate of this study indicates that overweight and obesity are common problems among the population. According to the 2016 statistics of the World Health Organization (WHO), nearly 2.0 billion adults around the world were overweight, of which 34.0% were obese [2]. The eligible participants had a BMI of 30.35, which is classified as obese according to the WHO criteria [2]. Only a small number of males (n=9) participated in this study possibly because of the gender disparities in attitude toward the overweight problem. Although the waist–hip ratio was above the normal range in both genders (males vs females: 0.95 vs 0.90), women significantly felt unhappier regarding the overweight problem than men. This finding may possibly be attributed to the active participation of women in weight reduction programs to establish a better image and appearance after becoming slim. However, android obesity (apple-shaped) is becoming more common in males, and the visceral fat, which lies deep inside the abdomen surrounding the internal organs, affects vital organs such as the heart, liver, kidney, and the lungs [44], thus increasing the risk of cardiovascular disease, hypertension, diabetes, sleep apnea, colorectal cancer, and premature death [44-46]. Therefore, additional strategies are needed to encourage males to deal with their overweight problem actively.

The high compliance rate, high satisfaction toward the trial arrangement, and the low attrition rate indicate the feasibility of the trial. These indicators also reflected a high motivation among the subjects (groups 1 and 2) who participated in the trial. Nearly all participants (97%) said that they would consider recommending this therapy to others.

Majority of the subjects had eating-out habits at least once per day (83%), perceived themselves as a gluttonous person, and ate even if they were not hungry (48%). Acupoint selection was mainly guided by the principles to reduce excessive calorie intake and to promote waste excretion to achieve weight reduction. For example, the use of “stomach” could reduce the motility of the stomach, diminish the sense of hunger, and raise serotonin levels, thereby suppressing appetite [13,15,35]. “External nose” is also called “hunger point” and “appetite control point,” which could heighten satiety and curb appetite [34]. “Forehead” could inhibit the central nervous system and increase satiety [33]. “Large intestine” can promote excretion [33], “endocrine” regulates endocrine function [33], and “shenmen” can reduce anxiety associated with weight loss and suppress the appetite of the subjects [13,15,35].

In general, the subjects in groups 1 and 2 achieved better therapeutic effect in certain anthropometric indices before meals than the waitlist controls. These findings are in accordance with the study conducted by Kim et al [17] who also demonstrated that AA is an effective intervention to reduce weight and BMI, decrease the sensation of hunger, and increase satiety of a person. Auricular stimulation modulates the hypothalamic neuronal activities associated with feeding, thereby curbing appetite [18,22]. Seeds for the administration of AA induce pressure to the skin according to the principle of neural reflexes similar to that of acupuncture [15] and influence appetite-related hormone peptides [16,47].

Although no significant differences in the pairwise comparisons between the 2 groups could be observed (*P*>.05), the decrease in body weight, BMI, body fat, visceral fat rating, and leptin level and increase in adiponectin level were more notable in group 2 before and after the intervention. The effect size of group 2 was also larger than that of group 1, indicating that the effect is substantial according to Cohen terminology [48]. Taking a conservative approach, a medium effect size with a sample size of 32 subjects/arm could be considered in future studies to detect 5% level of significance with 95% power.

Smartphones and wireless devices as mobile health have been widely used in recent decades to improve health outcomes. Previous studies have demonstrated the effectiveness of using smartphone apps as a useful tool to self-regulate diet for weight loss in patients with overweightness or obesity [49-53]. A meta-analysis on 11 studies indicated that reminder systems could significantly increase patient adherence to treatment [54]. Therefore, the daily reminders sent out to the subjects were hypothesized to increase their compliance to perform self-pressing, thereby achieving adequate acupoint stimulation. However, the absence of significant difference in pressing compliance between the 2 groups might indicate that the subjects in group 1 were independent and self-disciplined enough to follow the instructions and perform self-pressing as instructed even though no reminders were sent to them.

In general, the subjects in group 1 had higher perceived usefulness of the information booklet than those in group 2 mainly because the booklet was the only source of written information related to the therapy delivered to them. Subjects in group 2 expressed that the smartphone app was useful and were especially impressed by the daily reminders for performing self-pressing, the ear diagram indicating the locations and functions of the 6 ear points, and ear pressing method demonstrated in the video scripts. The stronger therapeutic effect observed in the protocol of group 2 might be because of the additional support, encouragement, and subjects’ interactions when using the app.

Adiponectin and leptin are 2 major hormones derived from adipose tissue (fat) [55]. Adiponectin controls body weight by regulating food intake and sensitizing insulin action in body tissues [22,56]. The adiponectin level is inversely correlated with body mass, especially visceral fat and metabolic health [22,57]. Weight reduction increases the serum adiponectin concentration, resulting in a decrease in leptin levels [29]. The adiponectin/leptin ratio in patients with obesity and diabetes is significantly lower than that in patients without obesity [58]. The elevated level of adiponectin also has a positive health impact because of its anti-inflammatory, antioxidant and antiatherosclerotic properties [59].

By contrast, leptin secretion may be an important mechanism, in which adipose tissue sends signals to the hypothalamic nuclei that the body has stored enough fat and thus no longer requires food intake [5]. Previous evidence demonstrates that AA is associated with decreased leptin levels, which are associated with weight loss [5,16,22,60]. The measurement of leptin and adiponectin can serve as a surrogate marker of metabolic heath and indicate whether the treatment has effects on adipose tissue function and systemic metabolism. In this trial, correlation analyses indicate that a significant correlation exists among the anthropometric indices and the leptin concentration (ng/mL) after the intervention. However, the changes of these biomarkers (leptin and adiponectin) among the subjects in group 2 are notable but not significant. Therefore, further investigations should be conducted before conclusive results of the pathway can be drawn.

Only 2 male subjects felt embarrassed because of the adhesive tapes placed on the auricles. No specific adverse effects arising from the therapy were observed, except 2 participants who reported having mild skin irritation on the ears because of the adhesive tapes used for positioning the experimental objects in place. The most tender acupoint felt by the subjects was “stomach,” followed by “forehead,” “endocrine,” “external nose,” “shenmen,” and “large intestine.” According to the auricular diagnosis system, the areas of the auricle with heightened tenderness upon touching correspond to specific areas of the body where some pathological conditions exist [61,62]. Applying seeds may induce physical pressure on the ear acupoints and cause tenderness, especially in cases with disequilibrium of the bodily functions (in this case, overweight problem) corresponding to specific acupoints. The tenderness on the reflective acupoints experienced by the subjects could be taken as a part of the treatment process rather than as adverse effects of auriculotherapy.

### Limitations and Recommendations of the Study

As this is a feasibility study enrolled with a small number of participants, therefore generalization of results is limited. A larger sample size with more male subjects should be considered in future trials to evaluate whether any gender disparities may have an impact on the treatment effect. Moreover, longer follow-up (a minimum of 3 months) after the therapy may be considered in evaluating the sustained treatment effect. The effectiveness of AA should also be evaluated in other populations, such as those with metabolic syndrome or children with obesity, so that their overweight problem can be tackled using a self-manipulated and nontraumatic approach.

### Conclusions

The high compliance rate, high satisfaction toward the trial arrangement, and low attrition rate indicate that AA can be used to achieve weight reduction and applied in future large-scale studies. AA integrated with the smartphone app has a more notable effect than using AA alone in terms of the decrease in body weight, BMI, body fat, visceral fat rating, and leptin level and increase in adiponectin level. Larger sample size should be considered in future trials to determine the causal relationship between treatment and effect.

## Appendix

Multimedia Appendix 1 Screenshots of the Auricular Acupressure for Weight Reduction, V1 app.

Multimedia Appendix 2 Flowchart of the trial.

Multimedia Appendix 3 CONSORT-EHEALTH checklist (V 1.6.1).

Multimedia Appendix 4 Correlational analyses among anthropometric indices (postintervention).

Multimedia Appendix 5 CONSORT-EHEALTH checklist (V 1.6.1).

## Abbreviations

AA: auricular acupressure
BMI: body mass index
CV: coefficient of variability
iOS: iPhone operating system
WHO: World Health Organization
