# Supplementary material for: Self-Administered Auricular Acupressure Integrated With a Smartphone App for Weight Reduction: Randomized Feasibility Trial
Source: JMIR Mhealth Uhealth. 2019 May 29;7(5):e14386. doi: 10.2196/14386 (PMC6658225; doi:10.2196/14386)
Supplement: Multimedia Appendix 1 [file mhealth_v7i5e14386_app1.pptx]

## Slide 1
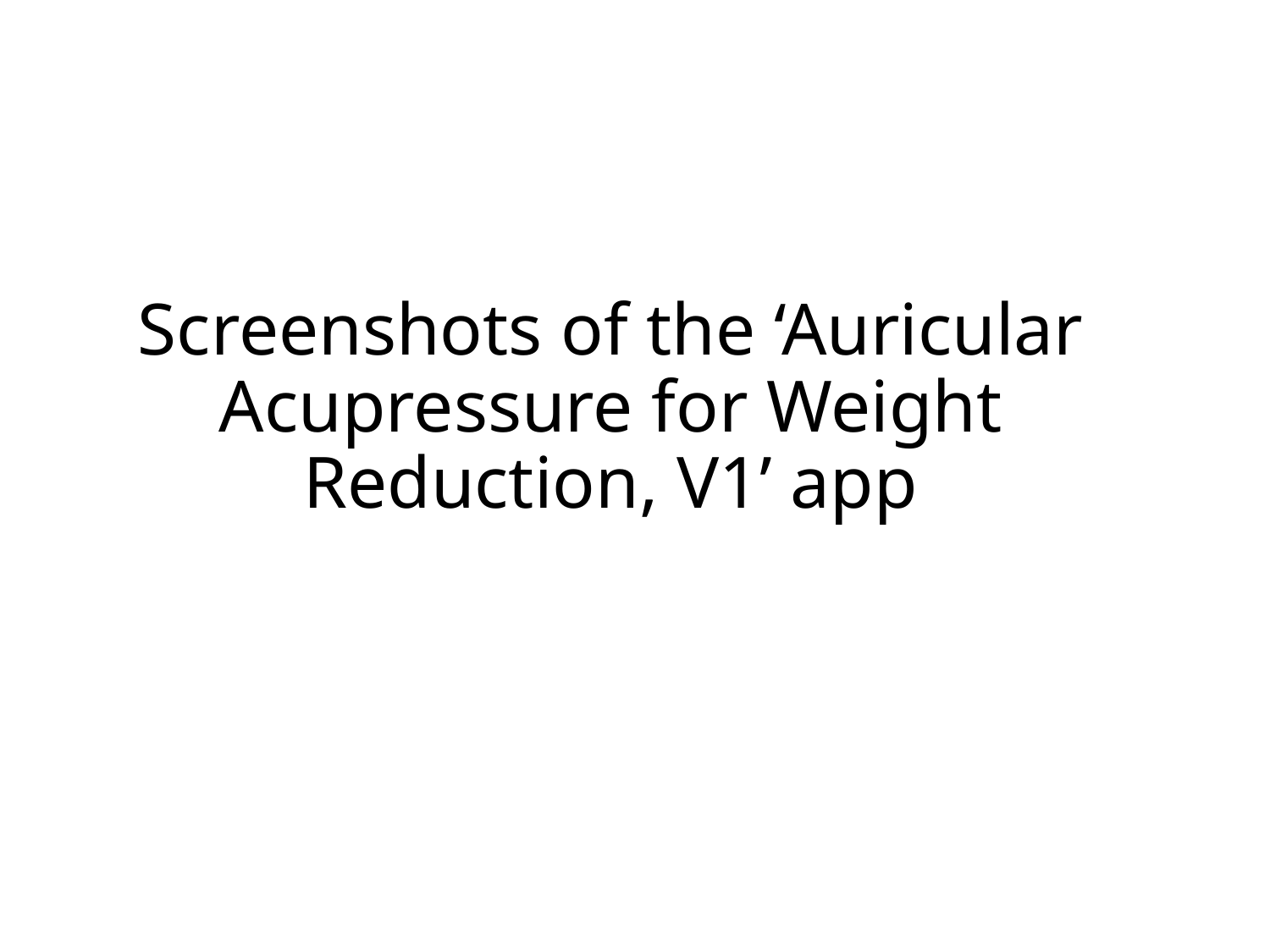

# Screenshots of the ‘Auricular Acupressure for Weight Reduction, V1’ app

## Slide 2
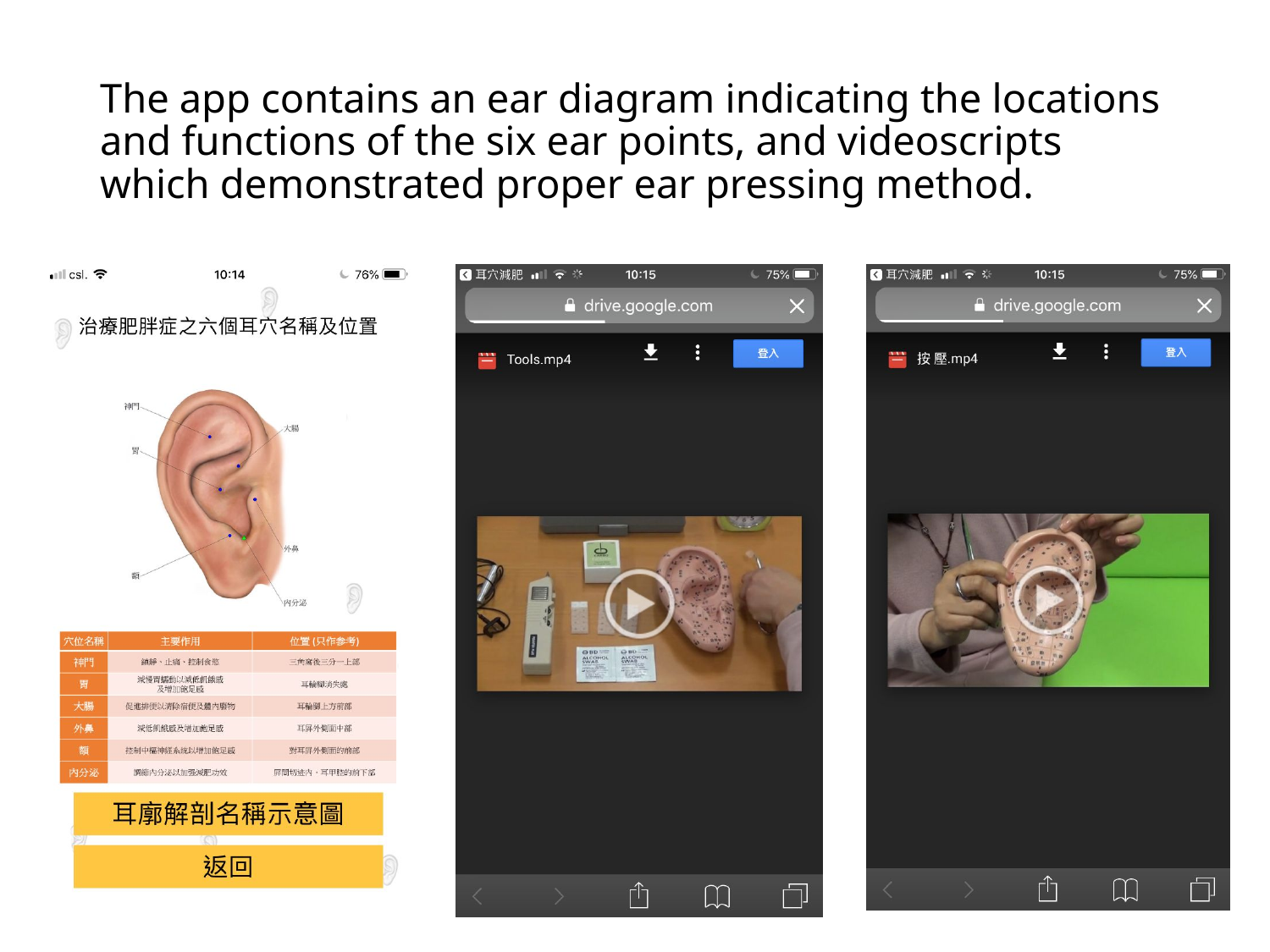

# The app contains an ear diagram indicating the locations and functions of the six ear points, and videoscripts which demonstrated proper ear pressing method.

## Slide 3
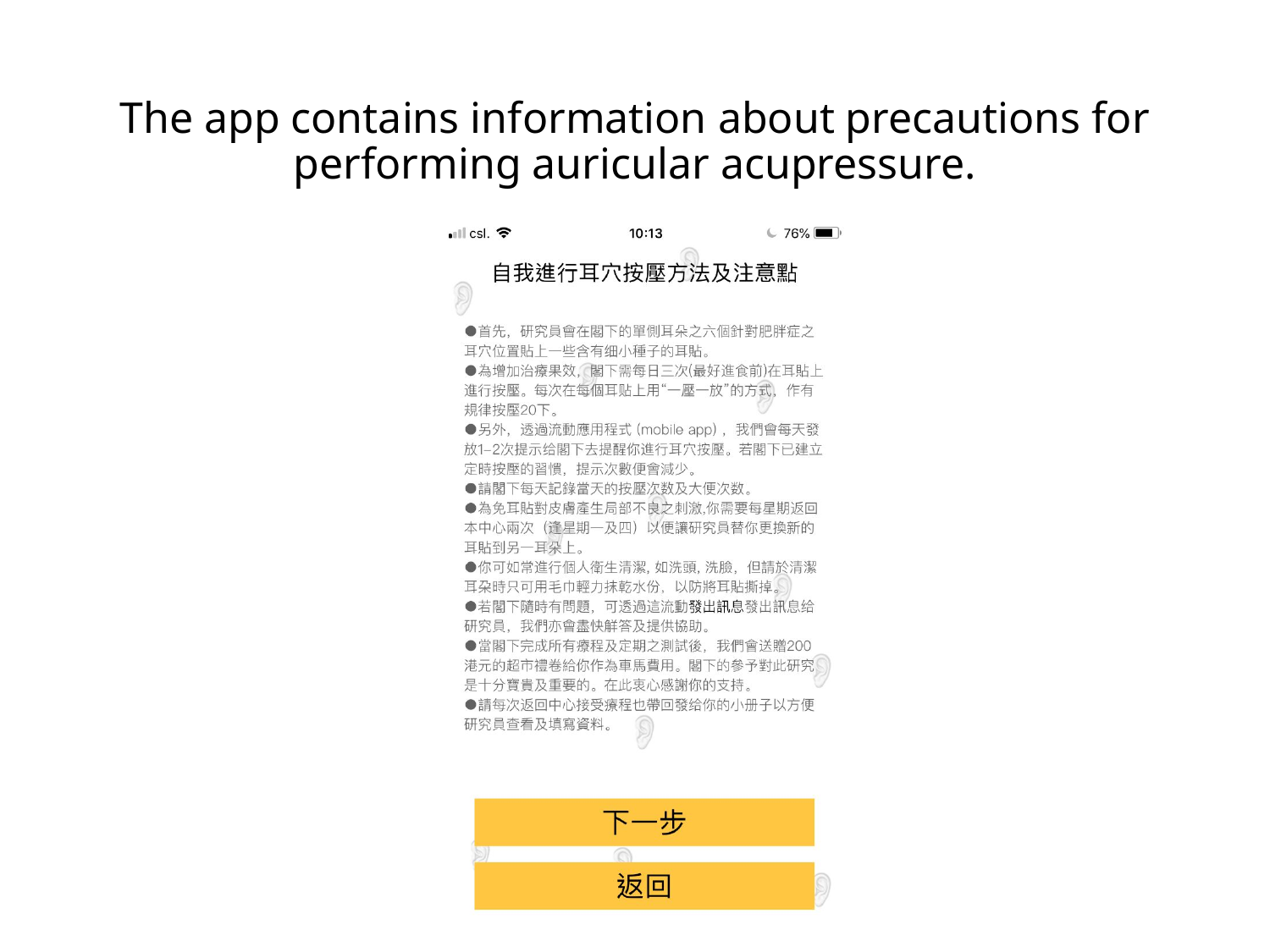

# The app contains information about precautions for performing auricular acupressure.

## Slide 4
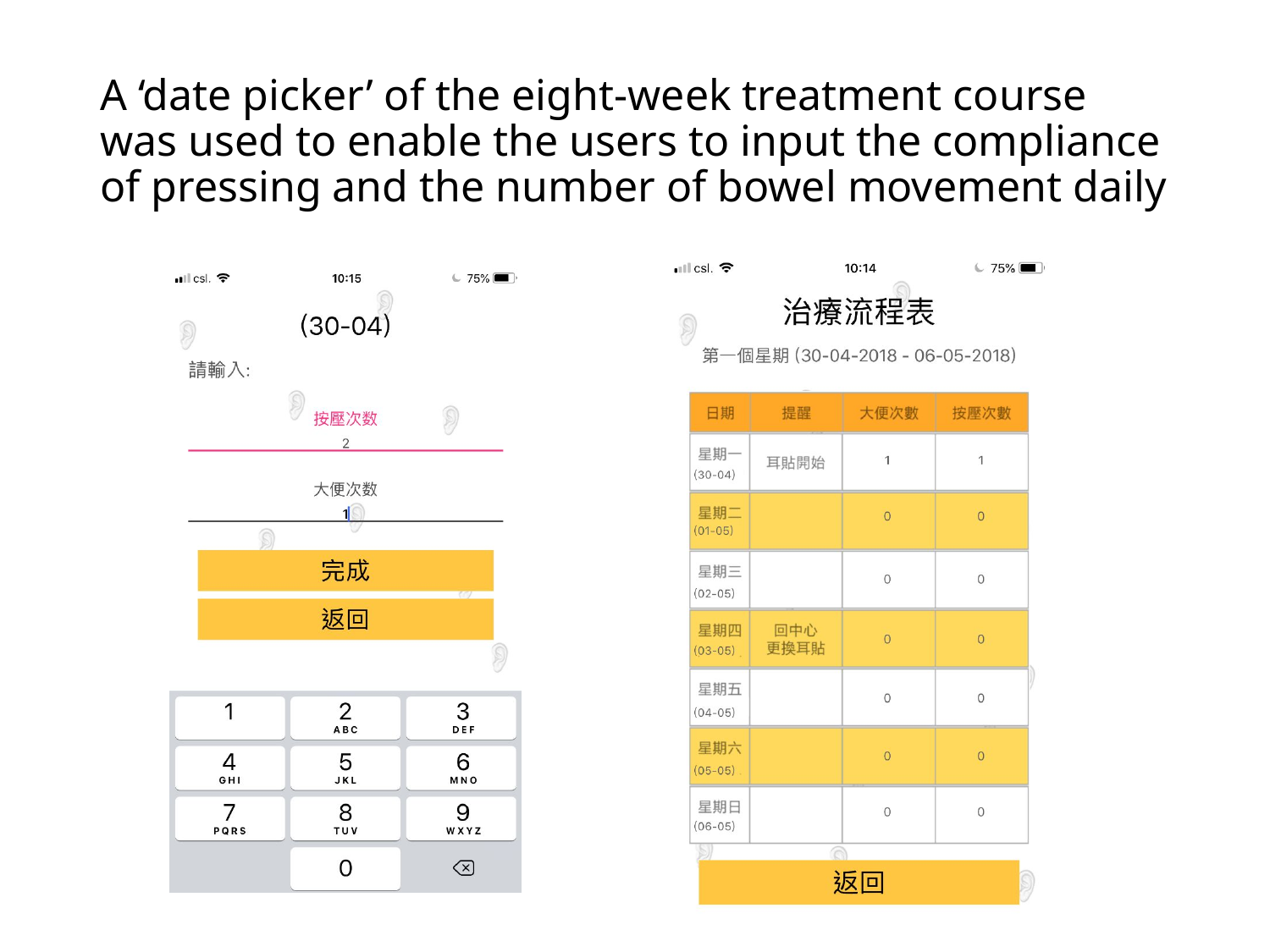

# A ‘date picker’ of the eight-week treatment course was used to enable the users to input the compliance of pressing and the number of bowel movement daily

## Slide 5
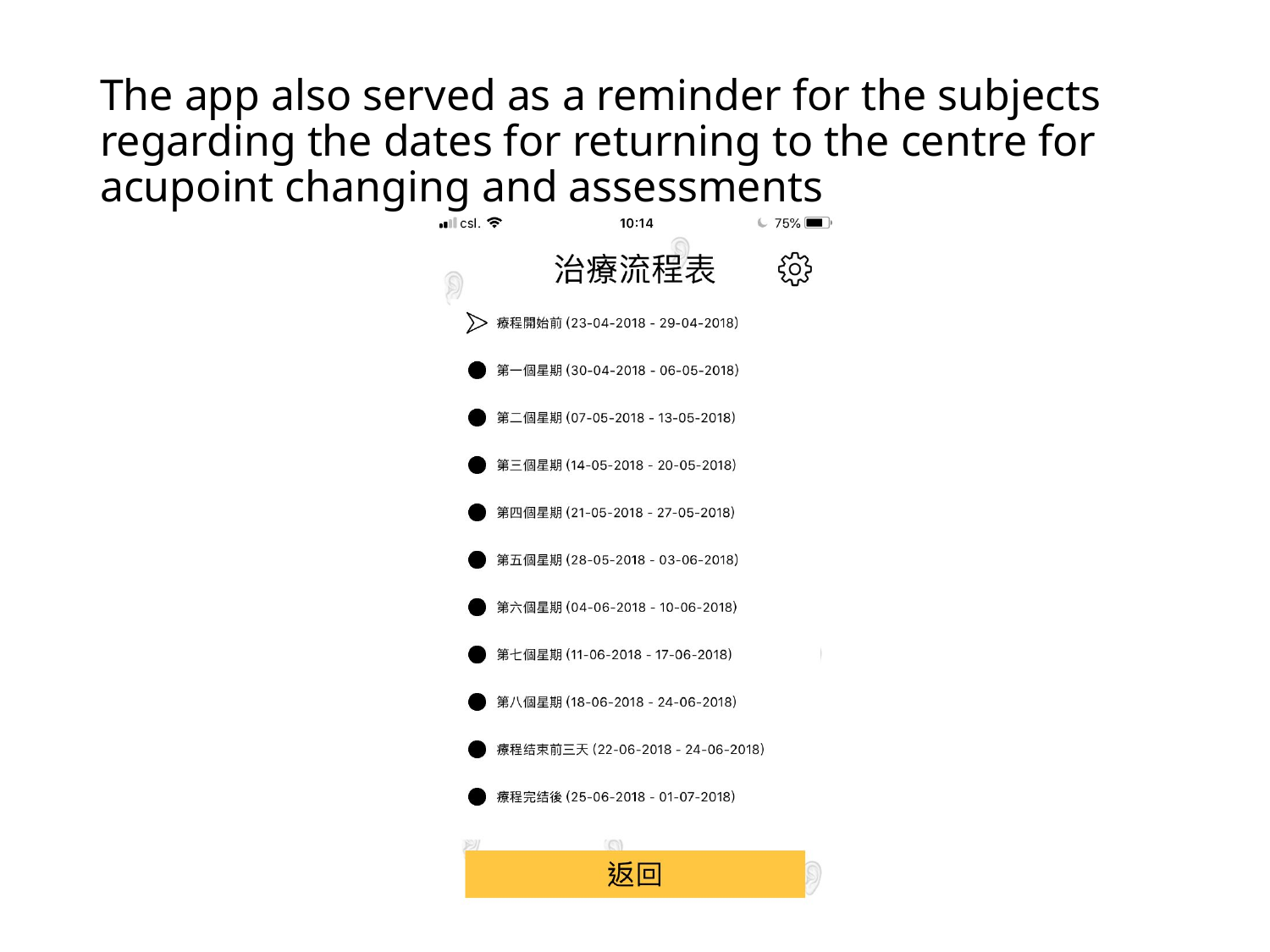

# The app also served as a reminder for the subjects regarding the dates for returning to the centre for acupoint changing and assessments

## Slide 6
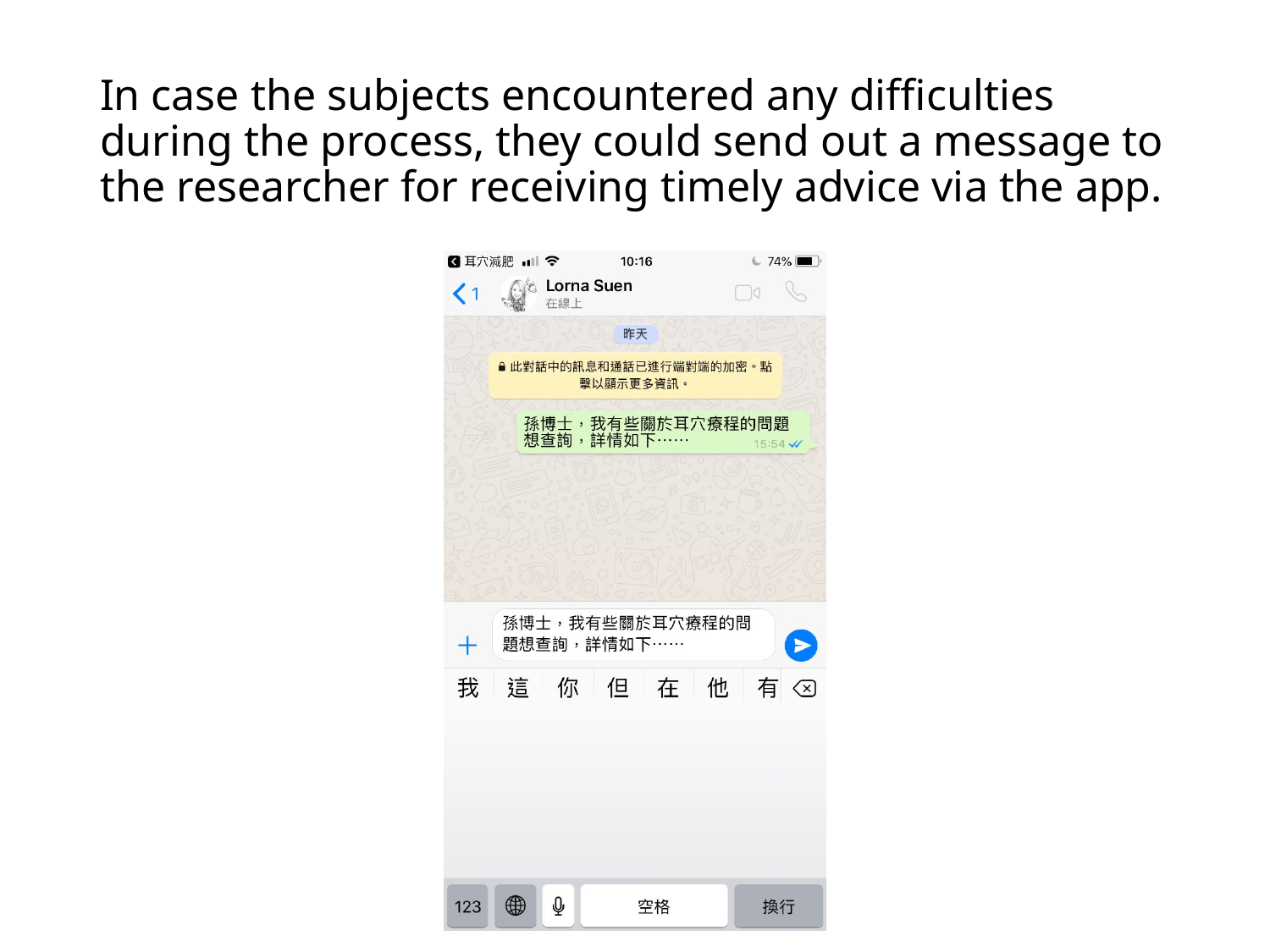

# In case the subjects encountered any difficulties during the process, they could send out a message to the researcher for receiving timely advice via the app.
