# Supplementary material for: Self-Administered Auricular Acupressure Integrated With a Smartphone App for Weight Reduction: Randomized Feasibility Trial
Source: JMIR Mhealth Uhealth. 2019 May 29;7(5):e14386. doi: 10.2196/14386 (PMC6658225; doi:10.2196/14386)
Supplement: Multimedia Appendix 2 [file mhealth_v7i5e14386_app2.docx]

Randomised (*n*=59)

PlaceboGroup 3

Placebo LAT and MAT

(n=35)

Group 2

Combined intervention

ed (*n*=102)

Flowchart of the trial

Assessed for eligibility (*n*=82)

Combined intervention

d for eligibility (*n*=120)

Excluded (*n*=23)

Not meeting inclusion criteria (*n*=20)

Planning to travel *(n*=3)

## Allocation

Group 1

Placebo LAT and

placebo MAT

*(n=*33)

*n*=4)

## Enrollment

## rollment

Group 1

Auricular acupressure

(*n*=19)

AT and

placebo MAT

*(n=*33)

Group 3

Waitlist control

(*n*=21)

## Allocation

Group 2

Auricular acupressure plus smartphone app

(*n*=19)

18 completed (85.7%)

Loss to follow up (*n*=3)

Health problem-eczema (*n*=1)

Family reasons (*n*=2)

## Post intervention

17 completed (89.5%)

Loss to follow up (*n*=2)

Family reasons (*n*=1)

Transport problem (*n*=1)

18 completed (94.7%)

Loss to follow up (*n*=1)

Family reasons (*n*=1)

## Analysis

GEE (*n*=19)

GEE (*n*=19)

GEE (*n*=21)
