# Supplementary material for: Self-Administered Auricular Acupressure Integrated With a Smartphone App for Weight Reduction: Randomized Feasibility Trial
Source: JMIR Mhealth Uhealth. 2019 May 29;7(5):e14386. doi: 10.2196/14386 (PMC6658225; doi:10.2196/14386)
Supplement: Multimedia Appendix 4 [file mhealth_v7i5e14386_app4.docx]

Multimedia Appendix 4 Correlational analyses amongst anthropometric indices (post-intervention) ^a^

| Measures | BMI | Body fat (%) | Body Water (%) | Muscle mass (kg) | Bone mass (kg) | Visceral fat rating | Waist cir. | Hip cir. | Waist-hip ratio | Leptin (ng/ml) | Adiponetin (ng/ml) |
| --- | --- | --- | --- | --- | --- | --- | --- | --- | --- | --- | --- |
|  |  |  |  |  |  |  |  |  |  |  |  |
| BMI | 1.000 |  |  |  |  |  |  |  |  |  |  |
| Body fat (%) | -0.764*** | 1.000 |  |  |  |  |  |  |  |  |  |
| Body water (%) | -0.775*** | -0.928*** | 1.000 |  |  |  |  |  |  |  |  |
| Muscle mass (kg) | -0.115 | -0.169 | 0.252 | 1.000 |  |  |  |  |  |  |  |
| Bone mass (kg) | -0.107 | -0.302* | 0.286* | 0.026 | 1.000 |  |  |  |  |  |  |
| Visceral fat rating | 0.571*** | -0.013 | -0.073 | -0.035 | 0.212 | 1.000 |  |  |  |  |  |
| Waist cir. | 0.858*** | 0.578*** | -0.628*** | -0.075 | -0.026 | 0.642*** | 1.000 |  |  |  |  |
| Hip cir. | 0.873*** | 0.728*** | -0.777*** | -0.167 | -0.104 | 0.453** | 0.807*** | 1.000 |  |  |  |
| Waist-hip ratio | 0.072 | -0.170 | 0.163 | 0.141 | 0.120 | 0.369** | 0.410** | 0.204 | 1.000 |  |  |
| Leptin (ng/ml) | 0.472** | 0.499*** | -0.458** | -0.046 | -0.169 | 0.117 | 0.359* | 0.484*** | -0.123 | 1.000 |  |
| Adiponetin (ng/ml) | 0.131 | 0.080 | 0.068 | 0.274 | -0.055 | -0.189 | -0.259 | -0.154 | -0.182 | 0.049 | 1.000 |

Abbreviation: BMI, body mass index; cir, circumference

^a^ Pearson product moment correlation (*r*)

* Correlation is significant at the 0.05 level (2-tailed)

** Correlation is significant at the 0.01 level (2-tailed)

*** Correlation is significant at the 0.001 level (2-tailed)
